# Analysis of peste des petits ruminants virus spread and the risk of its introduction into the territory of the Russian Federation

**DOI:** 10.14202/vetworld.2022.1610-1616

**Published:** 2022-07-07

**Authors:** Valeriy Alexandrovich Agoltsov, Dmitry Vasilievich Podshibyakin, Larisa Pavlovna Padilo, Oleg Yurievich Chernykh, Olga Mikhailovna Popova, Lyudmila Victorovna Stupina, Nataliya Victorovna Solotova

**Affiliations:** 1 Department of Veterinary Medicine and Biotechnology, Saratov State Agrarian University named after N.I. Vavilov, Saratov, Russia; 2 Scientific Research Institute of Technologies of Organic and Inorganic Chemistry and Biotechnology LLC, Saratov, Russia; 3 Department of Microbiology and Animal Virology, Kuban State Agrarian University Named after I.T. Trubulin, Krasnodar, Russia

**Keywords:** ecological niche, environmental factors, goat, linear regression model, maximum entropy model, peste des petits ruminants, sheep, species distribution

## Abstract

**Aim:**

This study examines methods to effectively control peste des petits ruminants (PPR), an emerging, highly contagious, transboundary disease that has been designated as a highly dangerous disease by the World Organization for Animal Health. Mathematical modeling was used as a predictive and preventive tool to assess the risk of PPR virus spread in the model area and the probability of its introduction into the territory of the Russian Federation.

**Materials and Methods:**

PPR risk assessment was performed by modeling the pathogen’s ecological niche by performing linear regression analysis in the geographic information system ESRI ArcGIS Desktop and maximum entropy methods using MaxEnt software. The territories of Bangladesh, China, and Algeria were used as model countries because they have the highest number of confirmed PPR outbreaks, as reported by the Food and Agriculture Organization of the United Nations from 2009 to 2020. The prepared global model of the PPR pathogen’s ecological niche was extrapolated onto the territory of the Far Eastern regions of the Russian Federation to assess the probability of virus introduction in that region.

**Results:**

Global model analysis showed that two factors exerted the highest influence on the spread of the PPR pathogen on a global scale: The minimum temperature of the coldest month of the year and the density of roads per unit area, which reflect the overall economic activity within a region. The highest risk of PPR spread was observed in areas with a minimum annual temperature of 16°C and road density of 5000 m/k^m^2^^.

**Conclusion:**

According to the model, areas with a dominant subtropical climate, where small livestock breeding is performed and where the average daily air temperature is >0°C throughout the year, are at the highest risk of PPR outbreaks. The risk of PPR spreading outside these areas is significantly reduced. Local extrapolation of the PPR ecological niche model demonstrates that the probability of epizootic development does not exceed 3–4% within the territories of the constituent entities of the Russian Federation adjacent to Mongolia and China.

## Introduction

Peste des petits ruminants (PPR) is a highly contagious, emerging transboundary disease of domestic sheep and goats and occasionally small, wild ruminants [1, 2]. PPR is known for its high morbidity (up to 100%) and mortality (up to 90%) rates, which lead to catastrophic financial losses for the small livestock industry globally. The direct costs of PPR epizootics alone can be as high as US $2 billion/year, not including the cost of vaccination campaigns targeted at susceptible livestock. According to existing estimates, approximately 1.7 billion susceptible farm animals (up to 80% of the world’s population) are at risk of PPR [3, 4].

PPR infection occurs primarily through the direct contact of healthy animals with sick ones through aerogenic and alimentary pathways because the PPR pathogen does not have significant resilience in the external environment. Hence, PPR spread may vary between areas depending on existing environmental and anthropogenic factors [2, 5]. In this regard, it is important to assess the risk of PPR spread, which is affected by a series of anthropogenic and biological factors in PPR-free areas.

This study aims to analyze the spread of the PPR virus in the model area and assess the probability of its introduction into the territory of the Russian Federation.

## Materials and Methods

### Ethical approval

Ethical approval was not required for this study because there was no animal or human subjects involved.

### Study period and location

The study was conducted in from July–December 2021 at Saratov State Agrarian University, named after N. I. Vavilov (Saratov, Russia).

### Datasets

To assess the impact of environmental and socio-economic factors on the risk of PPR spread, the World Organization for Animal Health and the Food and Agriculture Organization of the United Nations’ official data on outbreaks of this disease from 2009 to 2020 inclusive were used [6]. The global model of PPR virus spread is based on the outbreaks registered in Algeria, Bangladesh, China, and Mongolia. These countries were chosen due to the fact that over 93% (2715 out of 2897) of outbreaks reported worldwide during 2009–2020 were located in their territories. With its few outbreaks in 2016, Mongolia was added to the model area because it borders China and the Russian Federation.

To assess the influence of environmental factors on the PPR epizootic situation, a set of bioclimatic variables was utilized. Data on the average altitude above sea level in a raster format, with a spatial resolution of approximately 1 km^2^, were also used. These data were obtained from the WorldClim v.2.1 (https://www.worldclim.org) weather and climate database [7].

The number of susceptible farm animals (sheep and goats), the length of railways and highways, and the distance from seaports and airports were considered the socio-economic factors capable of influencing the spread of the PPR virus. Information about the livestock susceptible to PPR was obtained from the Gridded Livestock of the World Database 3 [8]. The length of railways per surface area of the territory was calculated using Vector Map Level 0 data [9]. The length of roads of all categories per surface area of the territory was obtained as a set of raster data with a spatial resolution of approximately 8 × 8 km from the GRIP global roads database [10].

The location data of airports and seaports, which were considered to be sources of infected animal import and obtained from the online databases Mile High Club v.4.1.0 (https://github.com/nvkelso/mile-high-club) [11] and High Seas v.4.0.0 (https://github.com/nvkelso/high-seas) [12]. All explanatory variables presented in raster format were reduced to a general spatial resolution of 1 × 1 km.

To extrapolate the resulting global model to the territory of the constituent entities of the Russian Federation adjacent to Mongolia and China, the data on existing communication routes in these territories obtained from the OpenStreetMap project with the NextGIS service were used [13]. Information on the number of susceptible animals in the target regions of the Russian Federation was obtained from the official publicly available source of the Russian Federal State Statistics Service [14].

### Statistical analysis and modeling methods

The significance of environmental and socio-economic factors in the spread of the PPR pathogen in the model area was assessed by constructing linear regression models using the exploratory regression tool, which is part of the ArcGIS Desktop (https://www.esri.com/en-us/ arcgis/ products/arcgis-desktop/resources) geographic information system. In this analysis, information about the localization of registered outbreaks of the disease was used as a dependent variable and the combination of socio-economic and environmental factors was used as explanatory variables in the linear regression equations. Independent variables related to models that passed the ordinary least squares (p < 0.05) and multicollinearity (variance inflation factor <3.5) tests were used for modeling of the PPR pathogen nosoarea using the maximum entropy method in MaxEnt v.3.4.4. (https://github.com/mrmaxent/Maxent) [15,16] Thus, five environmental and four socio-economic factors were chosen to build a model of the pathogen’s ecological niche (Table-1).

**Table 1:** Environmental and socio-economic factors used as explanatory variables in modeling the ecological niche of the PPR pathogen.

Name of the variable	Description of the variable	Units of measurement
bio_2	Mean diurnal range (mean of monthly [maximum temperature – minimum temp])	°C
bio_6	Minimum temperature of the coldest month	°C
bio_8	Mean temperature of wettest quarter	°C
bio_13	Precipitation of wettest month	Millimeters
bio_17	Precipitation of driest quarter	Millimeters
Goats	Goat population density	Total number of goats per km^2^
Railroads	Density of railways	m/km^2^
Roads	Density of roads	m/km^2^
Sheep	Sheep density	Total number of sheep per km^2^

PPR=Peste des petits ruminants

In the present study, modeling was conducted in ten replications of 10,000 iterations each, with the proportion of cross-validation data at 25%. The visualization of the cartographic data was carried out using ArcMap GIS Desktop (https://www.esri.com/en-us/arcgis/ products/arcgis-desktop/resources). The construction and classification of the database of outbreaks of PPR were carried out in MS Office Excel 2016 (Microsoft, USA).

## Results

The constructed MaxEnt model demonstrated a high predictive ability. The average test area under the curve of the receiver operating characteristic was 0.886 ± 0.008, which can be considered a sufficient indicator [17]. The relative importance of the individual factors (variables) used in the construction of the model after the permutation test is presented in Table-2. The obtained data indicated that the greatest influence on the distribution of the PPR outbreaks was exerted by the minimum values of the temperature of the coldest month of the year (variable: bio_6) and the density of roads (variable: roads). It is noteworthy, however, that the number of susceptible animals, especially sheep, played an insignificant role.

**Table 2:** Relative significance of selected factors (variables) for the spread of the PPR pathogen in the MaxEnt model.

Variable	Importance, %
Roads	30.6
Goats	1.7
bio_6	62.3
bio_13	1.1
bio_17	0.3
bio_2	2.6
bio_8	0.5
Sheep	0.3
Railroads	0.5

PPR=Peste des petits ruminants

**Figure-1:**
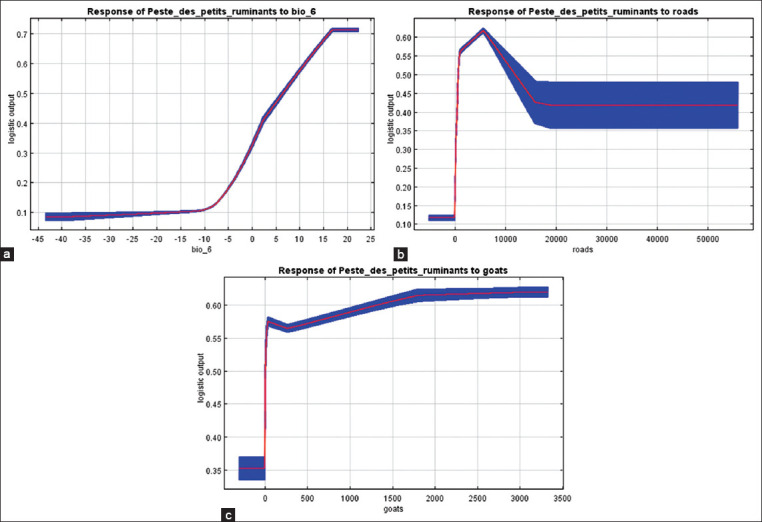
Response curves for variables that have the greatest impact on the risk of peste des petits ruminants spread: (a) Minimum temperature of the coldest month of the year, °C, (b) Density of roads, m/k^m2^, and (c) Density of goats, head/k^m2^.

The influence of the most significant variables in a MaxEnt model, as well as the number of goats at risk of PPR outbreak, is presented in the form of marginal response curves (Figure-1). In Figure-1, the Y-axis shows the relative probability of a PPR outbreak, calculated using a specific variable, provided that the values of other variables remain at the average level. The X-axis shows the values of the unit of measurement of variables according to Table-1. The red color shows the values of the variables calculated using ten repetitions of MaxEnt, and the blue color shows the standard deviation.

From the graphs, it is evident that the suitability of the territory for the spread of the PPR pathogen and, accordingly, the occurrence of an outbreak of the disease increased almost linearly from 10% to 71%, with an increase in the minimum temperature of the coldest month of the year from −10°C to +16°C. The optimal road density was equal to approximately 5000 m/km^2^. In this case, the suitability of the territory was estimated to be 62%. The optimal size of the goat population, when the probability of the presence of the PPR pathogen was 62%, was ≥1700 heads/km^2^. At the same time, with a smaller population size, this probability did not fall below 56%. The extremely sharp increase in this curve is noteworthy. Even with a much lower number of susceptible animals (several dozens of heads/k^m2^), the probability of the presence of PPR virus remained at the level of 56–57%.

**Figure-2:**
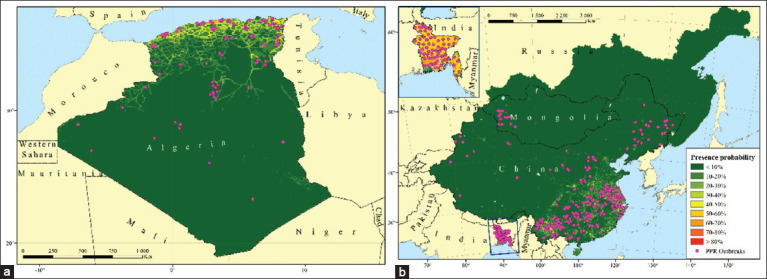
Maps of the probability of the presence of peste des petits ruminants pathogen in (a) Algeria, (b) Bangladesh, PRC, Mongolia, and Russia. [Source: Base map from https://gadm.org/index.html, retrieved on 24-05-2022].

The maps depicting the probability of the presence of the PPR pathogen in the territories that were used to build the model of its ecological niche are shown in Figure-2. The variables indicated in Table-1 were taken into account for preparing the maps. As can be seen from the results of the cartographic analyses, in Algeria (Figure-2a), the territories found to be most at risk of PPR outbreaks were located in the north of the country, where the probability of the presence of the PPR pathogen ranged from 30% to 70%. Bangladesh was the country seen to be most at risk of PPR outbreaks. There, in almost the entire country, the probability of the presence of the pathogen was 50%–70%. In China, the territories in the southeast had the highest probability of the presence of the PPR pathogen, which was in the range of 40–50% (Figure-2b).

**Figure-3:**
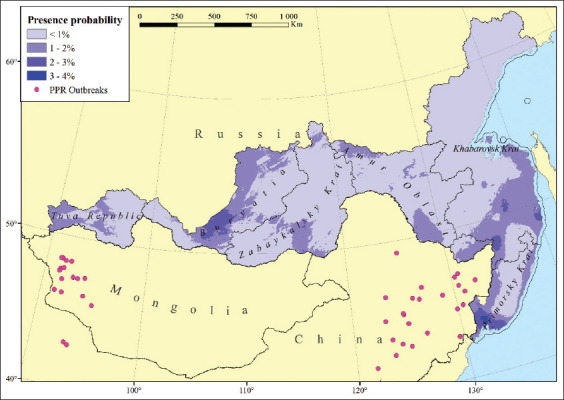
The likelihood of the peste des petits ruminants pathogen presence on the territory of the constituent entities of the Russian Federation bordering China and Mongolia [Source: Base map from GADM; https://gadm.org/index.html, retrieved on 24-05-2022].

Figure-2b also shows the likelihood of the presence of the PPR pathogen in the territories of the constituent entities of the Russian Federation directly bordering Mongolia and China. According to the scale used in this map, the probability of presence was <10%. However, on closer examination, it was obvious that this indicator was not homogeneous throughout this region (Figure-3).

The highest probability of the presence of the PPR pathogen was noted in the warmest part of this set of administrative entities, that is, in the South of Primorsky Krai, where it corresponded to 3–4%.

## Discussion

Environmental factors, such as air temperature, precipitation levels, and their change over time can affect the survival of the pathogen in the external environment. These factors also affect the likelihood of its transmission between susceptible individuals of a population and in the future, the possibility of forming a stable focus of this and other diseases [18]. Socio-economic factors, such as the density of the transport network and the remoteness from major transport hubs, determine the risk of pathogen spread to new territories. The previous studies have also suggested that an important role is played by the number of susceptible animals in a region and the variety of antiepizootic measures adopted by national veterinary services [19].

**Figure-4:**
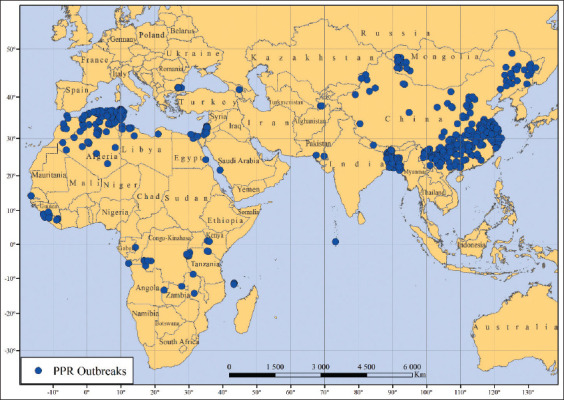
Outbreaks of peste des petits ruminants around the world, 2009–2020 [Source: Base map from GADM; https://gadm.org/index.html, retrieved on 24-05-2022].

**Figure-5:**
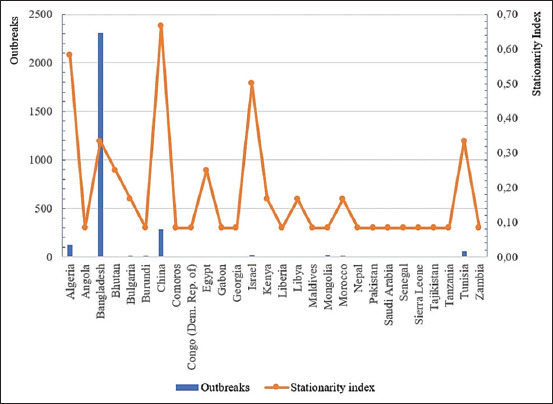
Number of peste des petits ruminants outbreaks and stationarity index in different countries, 2009–2020.

This study found that the combination of the factors considered in the global model can explain the distribution of PPR outbreaks in the world that occurred during 2009–2020 (Figure-4). Most of these outbreaks were documented in the subtropical climatic zone of the northern hemisphere, where the minimum temperature of the coldest month of the year does not fall below +10°C [13]. In the northern part of Algeria (Figure-2a), the subtropical Mediterranean type of climate prevails and the minimum temperature in winter ranges from +10°C to +12°C [7, 20]. In Bangladesh (Figure-2b), due to the tropical monsoon climate, the air temperature in January, which is the coldest month of the year, ranges from +16°C to +20°C during the day and is +10°C at night [7, 21]. In the southeastern part of China (Figure-2b), a subtropical monsoon climate prevails, with a minimum annual temperature of approximately 0°C [7]. The fact that favorable conditions for the spread of the pathogen have existed in the subtropical zone for a long time is also confirmed by the highest indicators of the stationarity index for countries such as Algeria, Bangladesh, Egypt, Israel, China, and Tunisia (Figure-5).

According to the constructed PPR distribution model, the probability of the presence of this pathogen outside the subtropical climate zone is greatly reduced, which is clearly visible on the maps of Algeria, China, and Mongolia (Figure-2a and b). It is noteworthy that no PPR cases were registered in the territory of Russia from 2009–2020 [6]. However, there is a hypothetical probability of its introduction from bordering countries where the disease is endemic. Seven regions of the Russian Federation share borders with Mongolia and China, namely, Amur Oblast, the Republic of Buryatia, the Jewish Autonomous Oblast, Khabarovsky Krai, the Tuva Republic, Primorsky Krai, and Zabaykalsky Krai (Figure-5). The climate of Tuva, Buryatia, and Zabaykalsky Krai is sharply continental, whereas Khabarovsky and Primorsky Krai and the Jewish Autonomous Oblast experience moderate monsoons. On the contrary, Amur Oblast has a transitional climate between continental and monsoon. The minimum temperature of the coldest month of the year in the southern border areas of these regions ranges from −35°C to −20°C [7]. This fact can explain the extremely low probability of developing PPR epizootics in these regions.

Outbreaks that have been registered outside the high PPR probability (≥30%) zones can be explained by human economic activities, for example, the transportation of infected livestock, livestock products, and equipment contaminated with the pathogen. This hypothesis can be confirmed by the fact that the second most important factor affecting the risk of PPR spread in the MaxEnt model was the density of roads in a local area. As inferred from Figure-2, the territories with a probability of the presence of ≥40% generally correspond with existing all-level road systems. In addition, since transportation in these regions is primarily through land and the transmission of the disease is the result of the contact route, the proximity of these areas to airports and seaports does not play a role.

## Conclusion

According to our modeling, the probability of introducing the PPR pathogen into PPR-free areas is primarily limited by the prevailing low minimum temperatures of the coldest month of the year. Therefore, the areas with a dominant subtropical climate, where small livestock breeding is developed and the average daily air temperature is above 0°C throughout the year, are most at risk of PPR outbreaks. The presence of a developed road network also contributes to a heightened probability for the occurrence of outbreaks. The risk of PPR spread outside these areas is significantly reduced. Local extrapolation of the PPR ecological niche model to the Russian Far East regions bordering Mongolia and China demonstrates that the probability of epizootic development in that territory does not exceed 3–4%.

The PPR virus distribution model presented in this study includes an insufficient number of available variables because more detailed data are not available from international databases, including those of the World Organization for Animal Health. In future studies, more variables should be considered during extrapolation, including local/regional factors such as animal migration, rearing methods, vaccination coverage, and vaccination effectiveness data, if such data are available. In addition, mathematical modeling methods and tools need to be developed further to enable more detailed models for the spread of PPR and other emerging infections.

## Authors’ Contributions

VAA and LPP: Designed the study, performed the modeling, and drafted the manuscript. OMP and LVS: Collected and analyzed the data and reviewed the manuscript. OYC and DVP: Performed statistical analysis and modeling, compiled the figures and graphs for the research, and drafted the manuscript. NVS: Drafted and reviewed the manuscript. All authors have read and approved the final manuscript.
